# The influence of estrogen deficiency on the structural and mechanical properties of rat cortical bone

**DOI:** 10.7717/peerj.10213

**Published:** 2021-01-13

**Authors:** Anna Shipov, Paul Zaslansky, Heinrich Riesemeier, Gilad Segev, Ayelet Atkins, Noga Kalish-Achrai, Stephen Weiner, Ron Shahar

**Affiliations:** 1 Koret School of Veterinary Medicine, The Hebrew University of Jerusalem, Rehovot, Israel; 2 Department for Operative and Preventive Dentistry. Centrum für Zahn-, Mund- und Kieferheilkunde, Charité - Universitätsmedizin Berlin, Berlin, Germany; 3 BAM Federal Institute for Materials Research and Testing, Berlin, Germany; 4 Department of Chemistry and Bar Ilan Institute of Nanotechnology and Advanced Materials (BINA), Bar-Ilan University, Ramat Gan, Israel; 5 Department of Structural Biology, Weizmann Institute of Science, Rehovot, Israel

**Keywords:** Estrogen, Disorganized bone, Rat, Lacunae, Ovariectomy

## Abstract

**Background:**

Post-menopausal osteoporosis is a common health problem worldwide, most commonly caused by estrogen deficiency. Most of the information regarding the skeletal effects of this disease relates to trabecular bone, while cortical bone is less studied. The purpose of this study was to evaluate the influence of estrogen deficiency on the structure and mechanical properties of cortical bone.

**Methods:**

Eight ovariectomized (OVH) and eight intact (control) Sprague Dawley rats were used.****Structural features of femoral cortical bone were studied by light microscopy, scanning electron microscopy and synchrotron-based microcomputer-tomography and their mechanical properties determined by nano-indentation.

**Results:**

Cortical bone of both study groups contains two distinct regions: organized circumferential lamellae and disordered bone with highly mineralized cartilaginous islands. Lacunar volume was lower in the OVH group both in the lamellar and disorganized regions (182 ± 75 µm^3^ vs 232 ± 106 µm^3^, *P* < 0.001 and 195 ± 86 µm^3^ vs. 247 ± 106 µm^3^, *P* < 0.001, respectively). Lacunar density was also lower in both bone regions of the OVH group (40 ± 18 ×10^3^ lacunae/mm^3^ vs. 47 ± 9×10^3^ lacunae/mm^3^ in the lamellar region, *P* = 0.003 and 63 ± 18×10^3^lacunae/mm^3^ vs. 75 ± 13×10^3^ lacunae/mm^3^ in the disorganized region, *P* < 0.001). Vascular canal volume was lower in the disorganized region of the bone in the OVH group compared to the same region in the control group (*P* < 0.001). Indentation moduli were not different between the study groups in both bone regions.

**Discussion:**

Changes to cortical bone associated with estrogen deficiency in rats require high-resolution methods for detection. Caution is required in the application of these results to humans due to major structural differences between human and rat bone.

## Introduction

Osteoporosis is a common metabolic bone disorder affecting millions of people worldwide with significant health, quality of life and financial consequences (Burge et al., 2007). Osteoporosis is characterized by decreased bone strength and increased risk of fracture, and is often manifested by low bone mass (Johnell & Kanis, 2005). However, osteoporosis is a multifactorial disease, and low bone mass alone does not account for increased skeletal fragility (Aspray et al., 1996). The vague term “bone quality” is often used to describe the ensemble of properties that influence the ability of bone to resist fracture. It is now believed that poor bone quality associated with osteoporosis is due to a combination of deteriorated bone material properties and micro-architectural disruption, leading to a tendency for bone failure (Aspray et al., 1996). The factors responsible for the deterioration of the material properties of osteoporotic bone include changes in the mechanical properties and mineral density of the bone material (Ma et al., 2008) and alterations in the size and density of osteocytic lacunae, which were shown to be associated with accumulation of micro-cracks (Vashishth et al., 2000).

Estrogen has several effects on bone metabolism, including down regulation of bone remodeling and maintenance of the balance between bone deposition and resorption (Riggs, Khosla & Melton 3rd, 2002). Postmenopausal estrogen deficiency has marked effects on both trabecular and cortical bone and plays a crucial role in osteoporosis and osteoporotic fractures. Estrogen deficiency increases bone turnover and may impair bone formation, resulting in net bone loss, while estrogen replacement therapy results in an increase of both cancellous and cortical bone volume in women suffering from postmenopausal osteoporosis (Khastgir et al., 2001; Wells et al., 2002).

Rats are one of the most frequently used animal models for studying the various aspects of osteoporosis (Jee & Yao, 2001). However, it has been previously shown that the basic structure of the cortical bone of rat femora differs substantially from human cortical bone (Bach-Gansmo et al., 2013; Shipov et al., 2013). Specifically, while the cortices of long bones of humans are primarily osteonal, rat cortical bone contains very few osteons and consists primarily of two distinct elements, which exhibit different structural, mechanical and compositional properties. One element consists of ordered circumferential lamellae, which are located in the endosteal and/or periosteal regions of the bone, and are presumably the product of normal bone modeling processes. The other structural element is characterized by a disorganized architecture, and is centrally located within the cortex, surrounded externally and/or internally by the circumferential lamellar bone tissue. Another structure unique to cortical bone of rats and mice are ‘islands’ of calcified cartilage which are present only in the disordered region and are most likely remnants of early endochondral ossification processes (Bach-Gansmo et al., 2013; Shipov et al., 2013).

Since bone is a hierarchical material, alterations that occur at the macro-, micro- and nano-scale can dominate its overall mechanical and structural properties. Such changes determine the so-called “bone quality” in healthy and diseased bone tissue. It is therefore important to obtain an insight into the structural and mechanical changes that occur at different hierarchical levels of cortical bone, when studying the effects of estrogen deficiency. Specifically, in rat cortical bone it is necessary to study the two distinct structural components (the disorganized region and the lamellar regions) separately, since these two tissues have markedly different structural and mechanical properties, and it is reasonable to assume that estrogen deficiency may affect them differently.

Cortical bone is known to be the major determinant of bone strength (Holzer et al., 2009; Ito et al., 2002). Nevertheless the majority of studies of osteoporosis are conducted on cancellous (trabecular) bone, while there are fewer studies investigating cortical bone. Furthermore, previous studies examining the effects of ovariectomy on cortical bone in rats reported conflicting results (De Carvalho et al., 2012; Miyagawa et al., 2011; Peng et al., 1997).

The aim of this study was to investigate the influence of estrogen deficiency on the structure and mechanical properties of rat cortical bone at the micro- and nano- scales in the two bone types: lamellar and disorganized cortical bone. This aim was achieved by comparing the structural and material properties of femoral cortical bone between ovariectomized and non-ovariectomized rats using nanoindentation, light microscopy, scanning electron microscopy, and synchrotron based micro-tomography.

## Materials and Methods

### Animals

Sixteen female Sprague Dawley rats were used in this study. They were divided into two groups: eight rats were ovariectomized at eight weeks of age (OVH group), and eight rats were left intact (control group). All rats were allowed access to standard rodent chow and water ad libitum, subjected to a 12-h light:dark cycle, and raised in individual cages in the same room. At the age of 8 months, all 16 rats were sacrificed by CO_2_ exposure.

The rats used here were part of another, much larger long-term study that investigated the effect of ovariectomy on cortical and cancellous bone using sequential injections of fluorochromes. Ethical approval to that study was given by the national committee for animal experimentation, approval # IL-12-03-047.

### Samples

Both femora were harvested for cortical bone analysis within two hours of sacrifice. Soft tissue was carefully removed from the bones, which were wrapped in saline-soaked gauze and stored at −20 °C until further testing.

### Light microscopy

**Figure 1:**
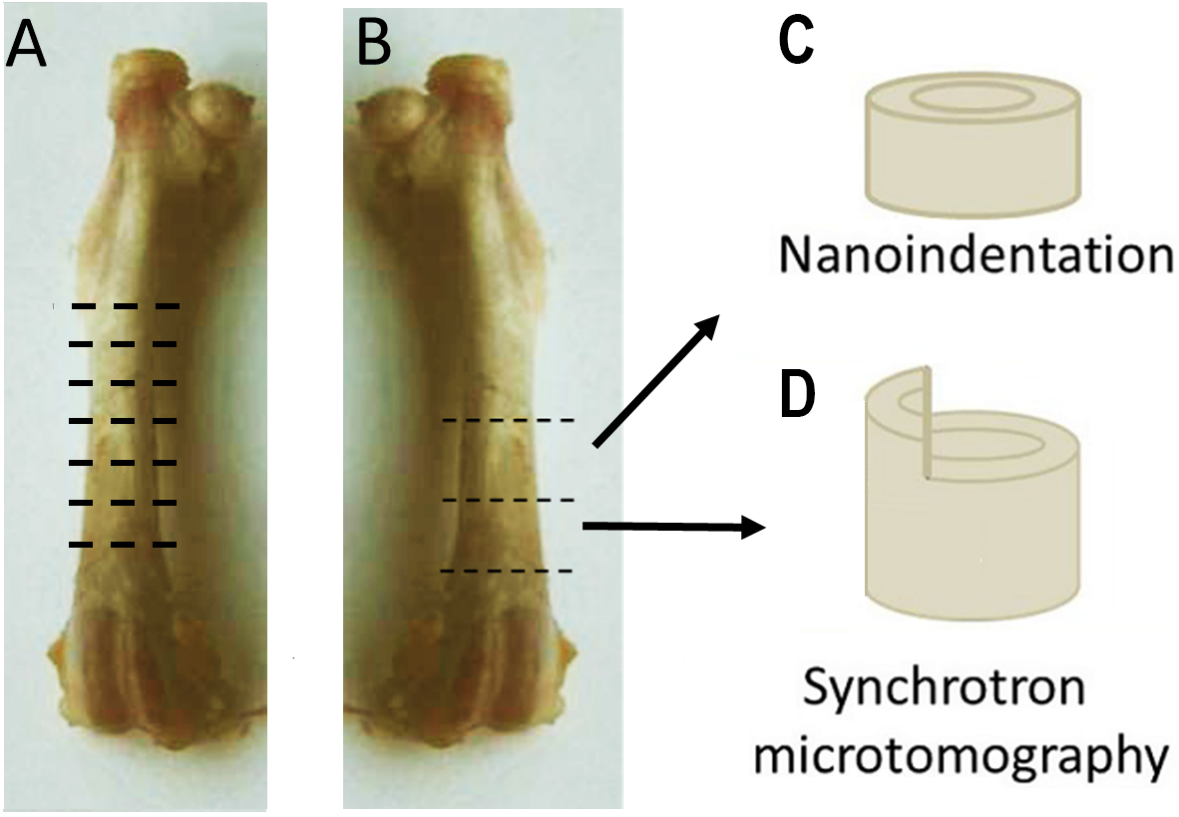
Sample preparation. (A) Structural features of the cortex were studied with light microscopy. Six ∼1 mm-thick cross-sectional samples were prepared from the diaphysis of the right femur, equally distributed along the length of the bone. (B) Two 2 mm-thick cross-sections of the distal shaft were prepared from the left femora. The proximal section was used for nanoindentation (C), While the distal for synchrotron based microtomography (D). Part of the proximal half of the circumference of each sample used for microtomography was removed, leaving a 1 × 1 × 1 mm protuberance above the circumference of the cortex.

Right femora of three OVH and three control rats were used for light microscopy studies. Six ∼1 mm thick cross-sectional (transverse) samples equally distributed along the length of the bone were prepared from the diaphysis of each femur (Fig. 1A) using a diamond-blade water-cooled saw (Isomet^®^ low speed saw; Buhler, Minneapolis, MN, Minneapolis, MN, USA). Each slice was marked such that its axial position as well as its proximal, medial and cranial aspects were clearly identified. The slices were ground using a grinding-polishing device (Minimet^®^1000; Buehler, Minneapolis, MN, USA) using a series of abrasive papers (increasing from 200 to 4000 grit), followed by polishing with cloths and 3 µm and 1 µm diamond paste. The polished bone surfaces were imaged using reflected-light microscopy (Olympus^®^ BX-51 microscope, Olympus, Japan). Images were captured using a 12.1 megapixel-resolution camera attached to the microscope (Olympus^®^ DP 71 digital camera, Olympus, Japan) and analyzed using image analysis software (ImageJ, NIH 1.47, USA).

### Synchrotron Micro-CT

Small (two mm long) cylindrical samples were prepared from the distal shaft of eight left femora of each group for multi-resolution imaging by monochromatic synchrotron radiation radiography available at the BAMline beamline of the BESSY II storage ring of the Helmholtz-Zentrum Berlin für Materialien und Energie (HZB). Part of the proximal half of the circumference of each sample was removed (using a dental burr), leaving a 1 ×1 × 1 mm protuberance above the circumference of the cortex (see Fig. 1B). The samples were scanned at two resolutions: the lower half of each sample, which contained the entire circumferences of the cortex, was scanned at a resolution of 4.35 µm effective pixel size, and the upper half of each sample, consisting of the protuberance, was scanned at an effective pixel size of 1.75 µm. The samples were mounted upright on the rotation stage of BAMline (Rack et al., 2008), and scanned in absorption mode using the double multilayer monochromator (DMM) centered at an energy of 23 keV. Tomographic datasets were obtained by rotating the samples by 180° at angular increments of 0.24°, obtaining 720∼750 projection (shadow) images per scan.

For void volume analysis an enhanced contrast imaging mode was used by in-line phase contrast for which 750 projection images were recorded at angular increments of 0.24° using an energy of 18 keV with a sample-to-detector distance of 25 mm. The radiograms were background-corrected by normalization with empty beam (flat-field) images, obtained intermittently, after subtraction of dark-current images. Reconstruction into 3-D datasets was performed using the PyHST reconstruction package from the ESRF (Grenoble, France).^11^

Structural 3-D data were analyzed using ImageJ (ImageJ, NIH, 1.47, USA) (Bolte & Cordelieres, 2006). In each bone sample 5–14 neighboring volumes of interest (VOI), ranging in size between 0.0022–0.039 mm^3,^ were selected, such that each VOI consisted exclusively of either lamellar bone or disorganized bone. Since the signal-to-noise ratio and acquisition parameters were optimized for density and size of our samples, excellent contrast between bone and void was obtained, and automatic thresholding using the Otsu algorithm was sufficient to binarize the images of each VOI, separating it into ‘bone’ (white) and ‘void’ (black) entities (Nuzzo et al., 2002; Shipov et al., 2013). Next, the volumes of all voids within the VOI were measured using the ImageJ plugin “Object Counter 3D” (Bolte & Cordelieres, 2006). Based on values of osteocyte lacunar volumes reported in the literature (Hannah et al., 2010; McCreadie et al., 2004; Shipov et al., 2013; Tommasini et al., 2012) and on the distribution of the sizes of voids in our study, voids within the bone matrix with volumes ranging between 75–625 µm^3^ were considered to be osteocytic lacunae, while voids larger than 625 µm^3^ were considered to represent vascular canals. The data were used to determine mean lacunar volume and lacunar density. Bone porosity was calculated for each VOI from the ratio of total void volume to VOI volume. Small voids, such as canaliculi, were not included in the analysis since their size is below the detection limit at the resolutions used in this study.

In order to allow valid comparisons between mean values of vascular canal volume (mm^3^) and vascular canal density (number of canals per mm^3^) determined in different-sized VOIs, normalization is required [see a detailed description in a paper by Shipov and colleagues (Shipov et al., 2013). Briefly, normalization can be achieved by two alternative approaches: The first approach assumes that when a VOI increases in volume the vascular canals in the original VOI simply extend into the larger VOI, therefore their volume increases, but their number does not change. The second approach assumes that the mean vascular canal volume in the larger VOI is the same as in the original VOI, but the number of canals increases. We chose a hybrid approach, in which we assume that both the number and volume of the vascular canals increases when the VOI increases, by the square root of the ratio between the VOI volumes, thus maintaining the volume fraction of the canals. We note that since the size of individual lacunae is relatively uniform and not affected by the differences in the size of the VOI, this normalization was not required when calculating mean lacunar volume and lacunar density.

Mineral density in different regions of the scanned bone was determined by calibrating attenuation values based on scans of 2 standardized phantoms (Bruker-CT, Kontich, Belgium) with known mineral density (0.25 g/cm^−3^ and 0.75 g/ cm^−3^). The density-attenuation relationship was assumed to be linear and the mean attenuation values of the two phantoms was used to create a tissue mineral density (TMD)-attenuation calibration curve. For each particular VOI, its TMD was calculated by determining the mean X-ray attenuation and using the calibration curve.

Volumes of interest were selected within the lamellar region, disorganized region and calcified cartilage islands.

Some of the specimens in the control group were previously analyzed in a different project (Shipov et al., 2013). These specimens were re-analyzed, using new VOIs to correspond to the VOIs in the OVH group.

### Mechanical testing: Nanoindentation

Three transverse sections from the left femoral mid-shaft of three rats in each group were used for nanoindentation experiments (Fig. 1B). The sections were dehydrated with increasing concentrations of ethanol, then embedded for eight hours in methylmethacrylate (MMA) which was polymerized in an oven at 60 °C. The embedded samples were cut with a diamond saw (Leica SP1600 saw-microtome) to a thickness of one mm, then ground and polished to create plano-parallel surfaces free of scratches and polishing damage.

Nanoindentation was performed using a scanning nanoindenter (TI950 Triboindenter, Hysitron Inc., Minneapolis, MN, USA) with a Berkovich diamond indenter tip. The nanoindenter was programmed to perform a scan of the surface, consisting of 2 lines 20 µm apart, with each line having an indent spacing of 10 µm, leading to a total of 120 indents in each sample. The following load function was used: maximum load of 5,000 µN, loading rate at 1,000 µN/s, holding at maximum force for 60 s and unloading to 1,000 µN at a rate of 400 µN/s, followed by a second holding time of 10 s and finally unloading to 0 µN at a rate of 200 µN/s. Measured properties included peak load, peak displacement and stiffness. The indentation modulus was calculated using the Oliver-Pharr method (Oliver & Pharr, 1992), based on the slope of the unloading curve in the region between 20% and 95% of the maximum load (Lewis & Nyman, 2008). The specimens in the control group were previously analyzed for a different project (Shipov et al., 2013) and used as a control in the current project.

### Scanning electron microscopy

**Figure 2:**
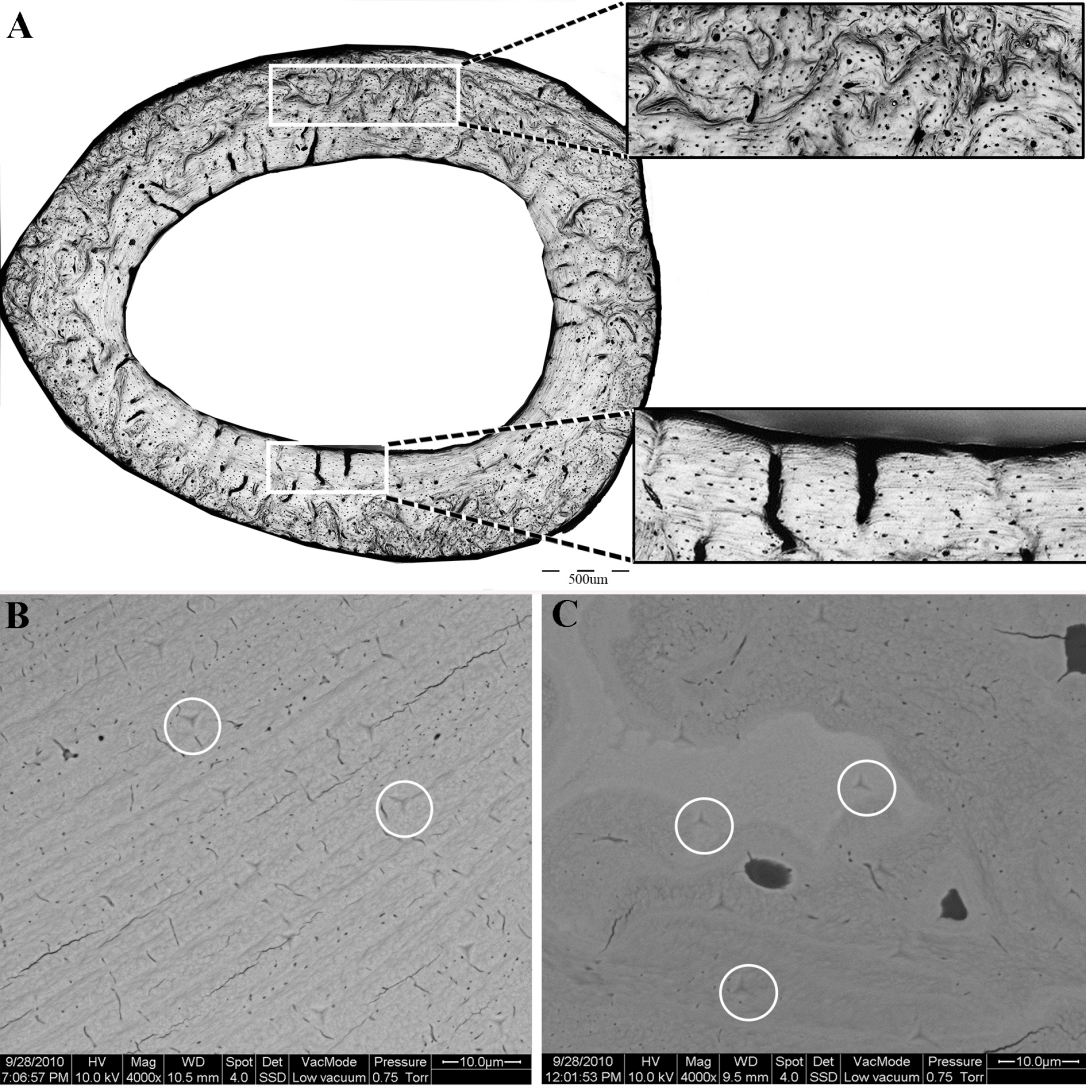
(A) Light microscopy, showing the two distinct regions of the proximal femoral cortex characteristic of both the OVH and control groups. Upper insert- disorganized bone. Lower insert—lamellar bone. (B, C) Scanning electron microscopy of the lamellar (B) and disorganized (C) regions of the bone. White circles delineate the indents of the nanoindentation experiment.

Following nanoindentation, the same samples were imaged (uncoated) in an environmental scanning electron microscope (ESEM, FEG Quanta 600, FEI, Eindhoven, The Netherlands) with a backscattered electron detector, using an acceleration voltage of 15 kV in low vacuum mode. Indentations within the bone were identified and categorized in terms of the structural element in which they were carried out (i.e., lamellar, disorganized or cartilaginous islands, Figs. 2B, 2C). Indents that were performed in PMMA or in the proximity of drying cracks or other voids were excluded from the analysis.

### Statistical analysis

Normality of distribution was assessed using the Shapiro–Wilk test. Normally distributed data are presented as mean ± SD. Continuous variables were compared between two groups using the Student’s *t*-test or the Mann–Whitney U-test based on data distribution. Continuous variables (e.g., lacunar volume, lacunar density etc.) that were measured multiple times in each bone were compared between the groups using the generalized estimating equations (since we had multiple measurements from the same bone, and therefore each one of these measurements could not be considered an independent measurement). For all tests, *P* < 0.05 was considered statistically significant. All calculations were performed using a commercially available statistical software package (SPSS 17.0 for Windows, Chicago, IL, USA).

## Results

### Morphology and structural properties

#### Light microscopy

All cortical cross sections, in both the OVH and control groups, clearly revealed two distinct microstructural regions (Fig. 2). One region consisted of circumferential lamellae and was located in the endosteal and/or periosteal aspect of the cross section. The other region had a non-lamellar, disorganized appearance and occupied the central part of the cortical cross-section. Highly mineralized islands of irregular shape, previously shown to consist of mineralized cartilage, appeared in the disorganized region (Bach-Gansmo et al., 2013; Shipov et al., 2013).

### Synchrotron based microtomography

**Figure 3:**
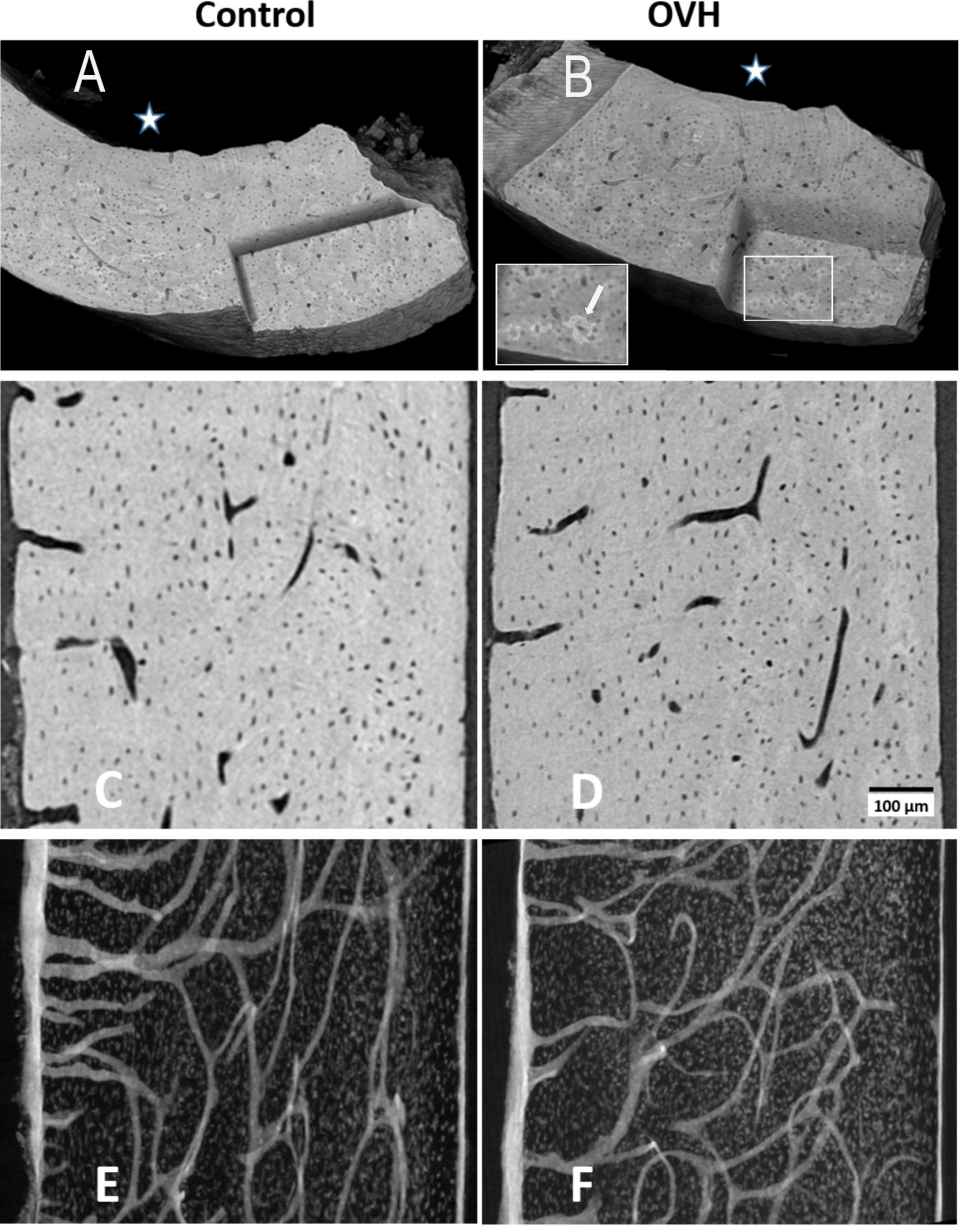
Typical views of synchrotron microCT data of control and of ovarictomized rats. (A and B) show 3D renderings of part of typical cortices of both groups, in which a corner is virtually cut out, to reveal parts of the internal bone structures. White star identifies the endosteal side of the bones. Cartilaginous islands within the bone can be identified by the higher mineral content (brighter color, insert B, white arrow). (C and D) show typical 3 µm thick tomography slices, where both osteocyte lacunae and vascular canals are clearly visible. (E and F) highlight the distribution of voids in axial slices along the bone shaft within a 125 µm thick slice. Vascular canal silhouettes appear to enter the bone at right angles to the endosteal bone surface (left hand side), and run both longitudinally and radially in the central bone region.

Synchrotron based microtomography scans were analyzed to compare mean lacunar volume, mean vascular canal volume and vascular canal density between the study groups for each bone type (disorganized and lamellar, Table 1, Fig. 3). Lacunar volume and density was significantly lower in the OVH group compared to the control group, in both the lamellar and disorganized regions (*P* < 0.001). Vascular canal volume was lower in the disorganized region in the OVH group compared to the same region in the control group, while no difference was found between the groups in vascular canal density, in both the lamellar and disorganized regions of the bone (Table 1).

**Table 1:** Results of synchrotron-based microtomography (Data presented as mean ± SD). Comparisons were made separately for the different bone regions between OVH and control groups.

Parameter	OVH		Control	
	Lamellar	Disorganized	Lamellar	Disorganized
Vascular canal volume (µm^3^)	159 ± 374 ×10^3^	62 ± 168 ×10^3^^**^	156 ± 374 ×10^3^	93 ± 314 ×10^3^
Lacunar volume (µm^3^)	182 ± 75^**^	195 ± 86^**^	232 ± 106	247 ± 106
Vascular canal density (canals/mm^3^)	129 ± 56^**^	241 ± 95^*^	134 ± 84	209 ± 91
Lacunar density (lacunae/mm^3^)	40 ± 18 ×10^3^^*^	63 ± 18 ×10^3^^**^	47 ± 9 ×10^3^	75 ± 13 ×10^3^
Porosity %	2.79 ± 1.57^*^	2.74 ± 0.77^**^	3.23 ± 1.06	3.83 ± 1.31

**Notes.**

* Significant (<0.05) differences between OVH and control.

** Significant (≤0.001) differences between OVH and control.

### Mechanical properties

Mechanical properties at different locations of the bone material were measured by nano-indentation. Indentation moduli of the cartilaginous islands in the control group were higher compared to those in the OVH group (33.40 ± 2.8 GPa vs. 29.8 ± 3.2 GPa, *P* = 0.03). There was no difference in the modulus of the lamellar region between the study groups (28.5 ± 3.0 GPa vs. 27.8 ± 4.3 GPa, *P* = 0.7), nor was there a difference in the modulus of the disorganized region between the study groups (30.1 ± 3.5 GPa vs. 28.1 ± 3.9 GPa, *P* = 0.17). Within each group (OVH and control), the cartilaginous islands had the highest modulus, followed by the disorganized region of the bone, while the lamellar region had the lowest indentation modulus.

### Mineral density

Tissue mineral density (TMD) was found to be similar in both groups in both the lamellar and disorganized regions. However, within each study group, significant differences were detected between the TMD of the two bone types (lamellar and disorganized), with the cartilaginous islands having the highest TMD (1.52 ± 0.05 g/cm^3^ in the OVH and 1.53 ± 0.04 g/cm^3^ in the control group, *P* = 0.363), followed by the disorganized region (1.40 ± 0.03 g/cm^3^ in the OVH and 1.41 ± 0.03 g/cm^3^ in the control group, *P* = 0.695) while the lamellar region had the lowest mineral density (1.37 ± 0.05 g/cm^3^ in the OVH and 1.36 ± 0.06 g/cm^3^, *P* = 0.134).

## Discussion

Here we show that ovariectomy in rats affects the structure and mechanical properties of the cortical bone. However, the changes we found were minor and their detection required high-resolution methods. The changes included an overall decrease in osteocyte lacunar volume and density, as well as lower vascular canal volume in the disorganized region of the bone material. These changes may affect the ability of cortical bone to respond to mechanical stimuli and maintain its health.

Osteocytes reside in lacunae embedded within the matrix of mature bone. It is widely believed that osteocytes play a crucial role in maintaining the health and mechanical function of bone by regulating the modeling and remodeling processes (Lanyon, 1993). Though extensive data regarding lacunar size are lacking, it has been shown that lacunae in mice become smaller with age (Heveran et al., 2018) and that smaller lacunae are associated with decreased response to mechanical loading (Hemmatian et al., 2018), indicating the importance of lacunar size in bone health. Previous studies comparing lacunar size in cortical bone of OVH vs. non-OVH rats had conflicting results. A study utilizing confocal microscopy did not find a difference in lacunar volumes in the proximal tibia six weeks after ovariectomy (Sharma et al., 2012), while others, using SRµCT, reported smaller lacunar volume in the endosteal region of the femoral cortical bone seven months after ovariectomy (Tommasini et al., 2012). We found that lacunae in OVH rats were significantly smaller in both the lamellar and disorganized regions. These morphological changes following OVH could affect the function of the osteocytic-canalicular system by impairing cell-to-cell communication and thus reducing the effectiveness of the osteocyte in mechano-sensing and damage repair, affecting the health of cortical bone in the long term.

Lacunar density is another parameter with major functional significance (Qiu et al., 2003; Vashishth et al., 2000). It has been shown that adequate numbers of osteocytes are essential for micro-damage sensing and removal (Busse et al., 2010). Furthermore, their density within the bone material positively correlates with anabolic activity of osteoblasts and the biomechanical quality of the bone, and negatively correlates with osteoclast activity and micro-damage sensing and accumulation (Ma et al., 2008; Qiu et al., 2005). In addition, osteocytic lacunae have been shown to affect crack propagation properties in bone and a lower density of osteocytes was found in patients with fractures compared to healthy controls (Qiu et al., 2003). Finally, estrogen deficiency is associated with osteocyte apoptosis in humans and rodents (Almeida et al., 2007; Tomkinson et al., 1997) while in sheep, it has been shown that in addition to decrease in lacunar density, the number of empty lacunae (lacunae that do not contain viable osteocytes) is almost twice as high in osteoporotic bone (Zarrinkalam et al., 2012). Our study demonstrates a ∼15% decrease in lacunar density in both the ordered and disordered regions of the cortical bone in OVH group. Since our analysis is based on synchrotron micro-tomography, we can only identify lacunae and determine their size and density, and cannot determine osteocyte viability. It is therefore possible that in the OVH group, there are not only fewer and smaller lacunae than in the control group, but also that some of these lacunae do not contain viable osteocytes due to increased apoptosis (Zarrinkalam et al., 2012). These effects may reduce the efficiency of the osteocytic network even further. Though lacunar mineralization in rats has so far not been reported, it is nevertheless possible that following apoptosis of osteocytes some of the lacunae become mineralized and merge with the surrounding matrix, as has been reported in aging human bone and in the zebrafish skeleton (Busse et al., 2010; Ofer et al., 2019).

All the above-mentioned alterations in osteocyte size, density and possibly viability, can potentially affect the ability of cortical bone to sense damage, maintain its health and allow accelerated damage accumulation, thus increasing bone fragility. It is plausible that in humans these changes occur over time and eventually become crucial as damage accumulates. However, such damage is less likely to become significant and detectable during a short-term experiment, or even during the entire life span of rats, which is much shorter than the life span of humans. This may account for the minimal differences in the mechanical properties observed between the OVH and control groups, and further emphasizes the shortcomings of rodents as a model for bone pathologies in humans.

Bone porosity is another characteristic of bone, which is a major determinant of its quality. The porosity of the disorganized region was higher in the control group, probably due to the larger volume of vascular canals found in this group. We note however, that cortical porosity was relatively low in all areas of both groups (between 2.8% and 3.8%). Sharma and colleagues (Sharma et al., 2018) reported that estrogen deficiency causes an increase in cortical bone vascular porosity in the proximal tibial metaphysis of rats, with enlarged vascular pores, but little change in tissue mineral density. However, studies reporting the effect of estrogen deficiency on the cortical bone of the rat (Sharma et al., 2012; Stern et al., 2018) have not investigated the effects separately in the two distinct zones of the bone (lamellar vs. disorganized) and the duration of these studies was short (weeks after ovariectomy), while the results reported here were found 6 months after OVH was performed.

Previous studies investigating the effects of ovariectomy on the stiffness of the bone material of rat cortical bone have reported inconsistent results. Some studies found decreased stiffness (Peng et al., 1997), while others found no change (Miyagawa et al., 2011) or even increased bone material stiffness following ovariectomy (De Carvalho et al., 2012). Other studies found a site specific or time-dependent effect, with initially superior mechanical properties of cortical bone in control rats, which disappears with time (12–28 weeks post OVH) (Peng et al., 1997). We found no difference in stiffness between the control and OVH groups in the lamellar and disorganized regions, and only mild differences in the material stiffness of the cartilaginous islands of the bone. We note however that this difference, although small, could affect mechanical properties such as fatigue resistance, which were not studied here.

One of the characteristics of post-menopausal osteoporosis in women is a disruption of the fine balance between bone deposition and resorption. It has been shown that in post-menopausal women bone resorption can increase by 90% while bone formation increases only by 45%, producing a net negative effect, thus decreasing its stiffness and mineral density (Garnero et al., 1996). Since the bones of small rodents such as mice and rats do not undergo significant cortical bone remodeling (Baron, Tross & Vignery, 1984; Erben, 1996), this may explain the lack of significant differences seen in mechanical properties and mineral density between the OVH and control study groups.

This study has several limitations. First, the number of animals used was relatively small, and larger groups could lead to strengthening of some of the statistical comparisons. Second, due to differences in bone structure between rats and human patients, the relevance of the results of this study with regard to humans is questionable and additional studies, evaluating these changes in a model animal that more closely simulates the human skeleton, are warranted.

In conclusion, we found that the effect of low estrogen levels on the cortical bone of rats is manifested by a lower density of osteocytic lacunae, which are also smaller, both in the lamellar and disorganized regions of the bone. These changes could lead to slow deterioration in bone quality, which might not be clinically manifested due to the short life span of rats. We note that the basic structure of rat cortical bone is markedly different from that of human cortical bone, since it does not undergo remodeling (while imbalance in the remodeling activity is considered the main cause of human osteoporosis), thus raising concerns as to the frequent use of rat cortical long bone as a model for human bone diseases.

##  Supplemental Information

10.7717/peerj.10213/supp-1 Supplemental Information 1 The database contains results of nanoindentation One column contains the group (ovariectomized vs. non ovariectomized), second column the sample number, the third the bone type.

10.7717/peerj.10213/supp-2 Supplemental Information 2 Lacunae and blood vessel volume

10.7717/peerj.10213/supp-3 Supplemental Information 3 Blood vessel volume within the sample Column A represents hormonal status (ovariectomized =2, non ovariectomized =1), column B represents the sample from which the measurements were made, column C represents the bone type (1=lamellar, 2= disorganized), column D represents the corrected vessel volume.

10.7717/peerj.10213/supp-4 Supplemental Information 4 Lacunar volume of all samples

## Additional Information and Declarations

### Data Availability

The following information was supplied regarding data availability: Data retrieved from the micro-CT scans and indentation are available in the Supplemental Files. Additional data is available at Morphosource and Figshare: http://www.morphosource.org/Detail/ProjectDetail/Show/project_id/1182. Shipov, Anna (2020): peerj-30209-blood_vessel_volume (1).xlsx. figshare. Dataset. https://doi.org/10.6084/m9.figshare.13204370.v1. Shipov, Anna (2020): peerj-30209-lacunae_and_blood_vessel_volume_and_number_of _lacunae_and_vessels_per_volume_and_porosity (1).xlsx. figshare. Dataset. https://doi.org/10.6084/m9.figshare.13204373.v1. Shipov, Anna (2020): peerj-30209-nanoindentation.xlsx. figshare. Dataset. https://doi.org/10.6084/m9.figshare.13204376.v1. Shipov, Anna (2020): peerj-30209-lacunae_nOVH_and_OVH_for_stats (1).xlsx. figshare. Dataset. https://doi.org/10.6084/m9.figshare.13204379.v1.
